# Secondary Surgical Treatment of Chest Wounds

**Published:** 1918-08

**Authors:** 


**The Secondary Surgical Treatment of Chest Wounds.** Professor TUFFIER.

The speaker believed that the surgical operations necessitated by the complications which follow wounds of the chest are relatively few. He divided them into the two following classes :

*Aseptic* complications, which include foreign bodies, hemothorax, pulmonary sclerosis, and ultimately pulmonary tuberculosis; and *Infectious* complications, which are constituted by purulent pleurisy, abscess and gangrene of the lungs.

I. He stated that foreign bodies should be extracted, after the usual process of localization, only when they cause functional troubles which can be definitely traced to their presence.

When the foreign body is aseptic, the phenomena attributed to it, the speaker believed, are really due to *pulmonary sclerosis.* In a great many cases, the functional troubles — pain, dyspnea, and difficulty in respiration — persist in exactly the same degree.

Chronic hemothorax may present either of two forms : an *extensive* effusion, or a *limited interlobar* effusion.

As regards *extensive hemothorax*, diagnosis is made by means of physical examination and the X-ray : successive punctures produce a reddish fluid, the distinctive characteristics of which the speaker has defined elsewhere; they consist in its incoagulability and indefinite reproduction after puncture. He said he had seen it last 12 to 15 months in spite of 27 punctures, with phases of slight fever, considerable debility of the patient, and without infection of any sort.

The seriousness of hemothorax arises not only from its long duration but from the fact that the corresponding side of the thorax collapses, retracts, and brings about a definite deviation of the spine with diminution of the respiratory field — the spirometer demonstrates dyspnea and a precarious general condition.

After a very long time the hemothorax gives way to a probable sclerosis, for the X-ray reveals a considerable thickening of the pleura, and physical examination shows a diminution of the vesicular murmur. It is generally situated at the base of the pleura and posteriorly.

The speaker said that he has not been able to do much to render the prognosis of this condition more favorable. At present, he treats all cases very early (within from seven weeks to two months), by pleurotomy — evacuation of the fluid — and wide separation of the ribs by means of his " separator ". If the chest wall is immobile during respiration and very resistant to the touch he decorticates it. If, on the contrary, the lung is still flexible, he merely opens the wound, thoroughly cleans out all the false membranes and closes the incision, whereupon the pneumothorax thus created heals definitely.

*Limited Interlobar Hemothorax.* The speaker has known of only 4 cases. The collection has shown under the X-ray, in all the cases, the signs of a hydatic cyst of the lung, i. e., a regular spheroid tumor about the size of a large tangerine orange, absolutely limited, with sharply defined edges, without sclerosis, without condensation of the pulmonary parenchyma, and without any apparent lesion of its periphery. After the puncture, the cavity collapsed to a great extent.

It would seem that in these cases the collection really becomes encysted, and only resorption is necessary to allow the lung to fill out the space occupied by the sanguinary effusion.

As regards a small number of cases of pulmonary *tuberculosis*, the speaker considers them due rather to suppuration, to hospitalization, and to strain, or even to individual predisposition. The tuberculosis often develops in the lung on the side opposite to the lesion. Operation which was performed in one case, was perfectly successful.

II. *Complications Due to Infection in Purulent Effusions of the Pleura.* The speaker and his colleague Depage advocate a method of treating cases of open purulent pleurisy, based on chemical disinfection of the wound followed by closure of the surgical incision, both in medical purulent pleurisy and in post-traumatic surgical suppurations.

*a) Treatment of pleural suppuration in unopened cavity* (7 cases). This comprises three steps :

Pleurotomy.
Chemical disinfection.
Closure.

*1st Step. Pleurotomy.* There are two kinds of cases to be considered, according to whether a pneumococcal purulent pleurisy or a non-pneumococcal suppurative pleurisy is in question.

In the first, is performed simple pleurotomy in the intercostal space. The incision is made at the point where the slope is greatest in the posterior axillary line. The " separator " is placed into the wound, enlarges the orifice, and allows a complete evacuation of the fluid and false membranes to be made.

In the second class of cases, a thoracotomy with resection of a single rib is preferable. It allows of the complete evacuation of all pathological exudates, the *de visu* exploration of the whole of the pleural cavity, and examination of the lung.

The exploration of the pleural cavity is important. Some cavities heal easily and the prognosis is good. Other cavities show deeper recesses which are difficult to fill. Here also the examination of the lung must be made with care.

Illuminating the cavity and examining it through the opening of a purulent pleurisy, it is found that the pleuro-pulmonary fold is much thickened at one point. This part of the pleural fold is excised and decorticated, revealing pulmonary suppuration completely isolated from the pleural cavity.

The last part of the operation is the insertion of Carrel tubes. Seven or eight of these tubes are placed into all the recesses, even the furthermost, in every direction, and fixed to the skin by a piece of adhesive plaster. In certain cases, the speaker has even strengthened them with a silver wire to ensure rigidity and prevent any displacement.

*2nd Step. Chemical Disinfection.* This is effected directly, by injecting Dakin's fluid into each tube every two hours by means of the rubber bulb. The bacteriological examination, which from the first informs us as to the nature and number of the infective agents, is made by taking a swab once a day from the discharge at three points : the surface, the tract, and the deep recesses.

After a period of from 9 to 30 days at the maximum, the pleural cavity becomes sterile and the surgical wound is closed.

*3rd Step. Closure.* As soon as sterilization has been effected, the incision is closed by the technic to be described later.

*b) Treatment of Fistular Purulent Pleurisy* (6 medical cases).

The purulent pleurisy has been opened, a fistula remains, and there is a persistent suppuration. These cases, which should have become extinct, are still numerous.

First of all, a bacteriological examination of the discharge is made by swabs taken from three points : 1) the deep part of the cavity; 2) the edges of the fistula; 3) the skin near the wound.

The nature of the discharge being thus established, the treatment is instituted. It comprises three steps, as in the procedure previously described :

1. Débridement and incision of the pleural adhesions;
2. Chemical disinfection ;
3. Closure, which is here quite different from the operative method.

*1st Step. Débridement and Loosening of the Pleural Adhesions.* After débridement of the wound and placing of the "separator", the pleural cavity is explored. The presence of false membranes on the surface of the lung, which is mobile, is then perceived. They may be removed by simple rubbing. The situation, number, and size of the false membranes are then observed, and if the cavity is infected it is only necessary to put in a series of Carrel tubes for the purpose of disinfection.

*2nd Step. Chemical Disinfection.* This must be carried out with extreme care. It is unfortunately badly supported in the case of bronchial fistulæ, and the Dakin injections frequently give access to everything which should be avoided. Throughout this disinfection the pulmonary inspection is continued daily and methodically, The pleural cavity is measured by the quantity of liquid which can be injected into it, and the rise and fall of the lung is calculated very easily by the difference in volume of the liquid which can be injected during inspiration and expiration.

As soon as the daily bacteriological examination of the secretions shows that sterilization has been obtained (one organism or less in four fields), the thoracic orifice is closed. A little before absolutely certain sterility of the wound is attained, however, it is prudent to suspend the antiseptic treatment and make cultures. For this purpose, after suppression of the tubes a dry dressing is applied to the wound and maintained in position for 24 hours. Three successive swabs are then taken from the depths of the wound, from the edges, and from the neighbouring skin. If all three remain negative the wound is sutured.

*3rd Step. Partial or Total Decortication.* When the incision in the chest wall has been dilated by means of the " separator, " the false membrane is seen and attacked at its periphery, that is to say, at the point of union of the lung and the thoracic wall. This part of the operation is difficult to perform, and the opening in the chest wall must be large enough to facilitate it. As soon as this disinsertion (for which a bistouri is used) is terminated, a long spatula made especially for the purpose is employed to separate lightly the false membrane. The lung is then seen to mobilize itself throughout the pleuro-costal groove

The pulmonary decortication is then performed, beginning at the outside and proceeding towards the interior, either by median, transversal, or longitudinal section, forming two divisions.

In certain cases this dissection can be made throughout the surface, and the lung is seen to expand out of its shell and fill the thoracic cavity.

The decortication may be complete, the surface of the lung remaining intact — in which case it is a matter not of pleurectomy but solely of *decortication*. A slight bloody discharge and the appearance of a few bubbles of air, in certain cases, demonstrate erosion of the parenchyma.

When this decortication is not possible the false pulmonary membrane must be removed *en bloc* from all points where it can be separated from the lung without too much tearing; and at the others, where it is too adherent, it must be thinned down.

If *absolutely firm adhesions* are found, such as one sees in a traumatic or medical pulmonary lesion, it is generally necessary to abandon them, as inter-pulmonary dissection is too dangerous. In other cases, they are found where nothing can explain the location.

Finally, if a bronchial fistula is discovered it should be closed by Lambert's method of closure.

If the pseudo-membrane of the *pleuro-parietal* fold is easily separable it can be removed; otherwise it may be left without any inconvenience.

*4th Step*. On termination of the operation the whole of the decorticated surface, after simple compression, remains dry. The fragments are mobilized, and the whole wound closed, either by two rows of stitches, the deeper of catgut and the superficial of silkworm, or by a single row through the skin and muscle.

If there is profuse sanguinary oozing the suture is incomplete. A light gauze dressing, which absorbs and evacuates the blood, is placed on the surface of the lung and the wound is partially closed.

The next day the dressing is removed and the preliminary stitches (previously made through the pleuro-parietal opening so as to allow of discharge) are tightened, and the suture is then complete.

Surgical tendencies are now exactly contrary to those which formerly prevailed; the thoracic cage is no longer placed before the lung in importance, and the lung must always be considered before the chest wall. The advantages are considerable for the functional future of the patient; the lung resumes its normal activity, whereas in the old methods of treatment everything tended to destroy it.

The final results are very interesting. When the cicatrization is complete one can examine the respiration by the measurement of each half of the thorax and by auscultation. In acute cases the final results give a normal respiration unless at the base of the lung. The diaphragmatic costal sinus disappears. In chronic cases the deformation of the thorax remains stationary after one has commenced the treatment of the patient. The respiratory capacity increases after cicatrization. The thorax of the side affected dilates.

